# Efficacy and safety of 0.6% sodium alginate solution in endoscopic submucosal dissection for esophageal and gastric neoplastic lesion: A randomized controlled study

**DOI:** 10.1111/den.13352

**Published:** 2019-03-18

**Authors:** Naomi Uemura, Ichiro Oda, Yutaka Saito, Hiroyuki Ono, Junko Fujisaki, Nobuyuki Matsuhashi, Ken Ohata, Naohisa Yahagi, Tomoyuki Yada, Masahiro Satoh, Hisao Tajiri, Masafumi Inomata, Seigo Kitano

**Affiliations:** ^1^ Kohnodai Hospital National Center for Global Health and Medicine Chiba Japan; ^2^ Department of Gastroenterology and Hepatology Kohnodai Hospital National Center for Global Health and Medicine Chiba Japan; ^3^ Endoscopy Division National Cancer Center Hospital Tokyo Japan; ^4^ Department of Gastroenterology Cancer Institute Hospital Ariake Tokyo Japan; ^5^ Department of Gastroenterology NTT Medical Center Tokyo Tokyo Japan; ^6^ Division of Research and Development for Minimally Invasive Treatment Cancer Center Keio University School of Medicine Tokyo Japan; ^7^ Department of Innovative Interventional Endoscopy Research The Jikei University School of Medicine Tokyo Japan; ^8^ Division of Endoscopy Shizuoka Cancer Center Shizuoka Japan; ^9^ Department of Research and Development Kaigen Pharma Co. Ltd Hokkaido Japan; ^10^ Department of Gastroenterological and Pediatric Surgery Oita University Faculty of Medicine Oita Japan; ^11^ Oita University Oita Japan

**Keywords:** endoscopic submucosal dissection, gastric neoplasms, randomized controlled trials, sodium alginate, sodium hyaluronate

## Abstract

**Objectives:**

Sodium alginate (SA) solution has characteristic viscoelasticity. We aimed to determine efficacy and safety of 0.6% SA for submucosal injection during endoscopic submucosal dissection (ESD) in patients with localized neoplastic lesion in the esophageal and gastric mucosa.

**Methods:**

We conducted a randomized controlled study at six major hospitals in Japan including 130 patients with endoscopically localized neoplastic lesion in the esophageal and gastric mucosa and eligible for ESD. Patients were randomly assigned to SA or 0.4% sodium hyaluronate (SH) group (control); ESD was performed using a submucosal injection of SA/SH. As a primary outcome measure, non‐inferiority of SA against SH was investigated using en bloc complete resection in ESD and formation and maintenance of mucosal elevation upon injection as an efficacy index. Adverse events during the study were evaluated as safety outcome measures. This study was registered with Pharmaceuticals and Medical Devices Agency (clinical trial no. 28‐277/2016‐18; clinical trial identification no. KP2013‐009_C001).

**Results:**

Efficacy rate of submucosal injection during ESD was 91.7% (55/60) and 88.7% (55/62) in the SA and SH groups, respectively, demonstrating non‐inferiority of SA against SH. Adverse events for which a causal relationship with submucosal injection solution could not be eliminated were noted in 8.2% (5/61) and 4.7% (3/64) in the SA and SH groups, respectively, but symptoms disappeared without treatment/after drug administration in both groups.

**Conclusions:**

In Japan, 0.4% SH is the only commercially approved formulation for submucosal injection during ESD. The study results may expand submucosal injection solution options in clinical practice.

## Introduction

In Japan, endoscopic resection is accepted as the optimal treatment for early gastrointestinal cancers when the possibility of lymph node metastasis is extremely low.^1^, ^2^ For performing endoscopic resection in clinical practice, endoscopic submucosal dissection (ESD) is currently the most common and promising surgical technique for en bloc complete resection.^3^, ^4^, ^5^, ^6^

For easier and safer ESD, it is important to maintain sufficient mucosal elevation for lesions and their surroundings during all ESD procedures. To facilitate this, various submucosal injection solutions, such as hypertonic saline, glucose, glycerin/fructose and sodium hyaluronate (SH), have been studied in addition to physiological saline.^7^, ^8^, ^9^

Among these solutions, 0.4% SH is useful and safe as a submucosal injection solution;^10^ it is widely used in ESD in Japan. Additionally, it is the only submucosal injection solution to be approved in Japan. However, SH is derived from an animal product, and there may be concerns about allergy as an adverse reaction. Matsui *et al*.^11^ reported that SH promoted cancer cell proliferation in a persistent wound‐promoted tumor mouse model; therefore, it should be carefully used in the case of lesions with a high persistence risk.

Sodium alginate (SA) exhibits high water retentivity and viscoelasticity and is used in clinical practice as a medication for peptic ulcer or as a hemostatic agent.^12^, ^13^, ^14^ Akagi *et al*.^15^ noted that 3% SA could be a novel submucosal injection solution during ESD. Kusano *et al*.^16^ improved the formulation of 3% SA and reported that 0.6% SA could form and maintain mucosal elevation better than 0.4% SH, which is widely used in ESD.

Therefore, in this study, efficacy and safety of 0.6% SA for submucosal injection during ESD in patients with localized neoplastic lesions in the esophageal and gastric mucosa were compared with those of 0.4% SH during ESD, as a control.

## Methods

### Patient selection

Of patients who had a neoplastic lesion localized by endoscopy in the esophageal and gastric mucosa from March 2017 to July 2017 and who were eligible for ESD treatment, those meeting all selection criteria (see the inclusion and exclusion criteria listed below) and who provided written informed consent after thorough presentation of this study were registered.

Patients aged 20–80 years with neoplastic lesion localized in the esophageal and gastric mucosa (tumor size, 5–20 mm) were included.

Exclusion criteria included SH hypersensitivity history; concurrent advanced malignant tumor; antitumor drug systemic administration; severe hepatic/renal/cardiovascular disease; lesion that had spread to the pyloric ring or esophagogastric junction; lesion with an ulcer; operative treatment history of endoscopic resection for the target lesion; anticoagulant administration; pacemaker use; pregnant, lactating or women desiring to become pregnant during the study; and patients deemed inappropriate as a subject of the study by the attending physician.

This study was conducted in accordance with the ethical principles of the Declaration of Helsinki, and good clinical practice was adhered to. The study protocol was reviewed by the institutional review board of each participating institution, and conducted after approval was obtained.

This study was registered with the Pharmaceuticals and Medical Devices Agency (clinical trial no. 28‐277/2016‐18, clinical trial identification no. KP2013‐009_C001).

### Concomitant medication

The use of anticoagulants and antiplatelet drugs was prohibited. However, for antiplatelet drugs taken for complications prior to providing written informed consent, administration was performed in accordance with the gastrointestinal endoscopy clinical practice guidelines for antithrombotic drug users.^17^ During ESD, the use of agents aimed at elevating mucosa, such as physiological saline or glycerin/fructose solution, was prohibited, except for the SA or SH. While mixing epinephrine and indigo carmine into submucosal injection solution was permitted, mixing of other drugs was prohibited.

### Study device

Sodium alginate was dissolved at a concentration of 0.6% (wt/vol) and was stored in a 20‐mL glass vial at 1–30°C. MucoUp^®^ (Seikagaku, Tokyo, Japan) was used as the control (0.4% SH) and was stored at 1–30°C. There was no upper limit for the usage volume of these submucosal injection solutions and the volume necessary to create a mucosal elevation was allowed. The volume per local injection was not predetermined and was left to the judgment of the attending physician.

### Study design

This study was conducted at six primary hospitals in Japan; patients were registered and allocated by a central registration method. Each patient was randomly assigned using a dynamic allocation method with a 1:1 allocation ratio for the submucosal injection solutions, with facility and lesion site as adjustment factors. This study was conducted as a single‐blind study on patients only. Lesion resection was performed using the ESD technique by a certified endoscopist or by an experienced staff member under the supervision of a certified endoscopist. In all facilities, endoscopes equipped with a water jet function were used. Regarding the primary outcome measure, we comprehensively assessed en bloc complete resection in ESD (en bloc resection with histopathologically negative resection margin) and the formation and maintenance of mucosal elevation during injection (number of additional injections to the same place when mucosal elevation had disappeared; Table 1). When the required additional injection was once or none, ratio of en bloc complete resection was defined as the efficacy rate and compared between the SA and SH groups. Pathological examination of resected specimens was conducted on consecutive sections (width, 2 mm) according to Japanese guidelines for handling esophageal and gastric cancer.^18^, ^19^

**Table 1:** Primary outcome measure

En bloc complete resection	Additional count	Total evaluation^a^
Complete	0	Excellent
	1	Good
Incomplete or not evaluable	≥2	Moderate
	―	Poor

a ^†^Total evaluation of the primary outcome measure was assessed by comprehensively evaluating en bloc complete resection and the number of additional injections during endoscopic submucosal dissection as the efficacy rate. Primary outcome measure (efficacy) was defined as the percentage of excellent or good criteria.

Secondary outcome measures included mucosal resection ease (extremely easy, easy, ordinary and difficult), mucosal elevation shape (steep, gentle elevation, no elevation and unable to determine), time required for mucosal resection and volume of submucosal injection solution used. Mucosal resection ease and mucosal elevation shape were subjectively evaluated by the attending physician during ESD.

For safety analysis, we recorded all adverse events prospectively that occurred in all patients in whom submucosal injection solution was used and evaluated the causal relationship with submucosal injection solution. Cases falling under the following criteria were defined as serious adverse events: (i) mortality; (ii) mortality risk; (iii) hospitalization for treatment or hospitalization duration prolongation; (iv) impairment (persistent or noticeable impairment/dysfunction); and (v) impairment risk.

In this study, when a patient experienced an adverse event, he/she was monitored until recovery or until the attending physician determined that monitoring was no longer necessary.

The follow up was set to 8 weeks after ESD to assess healing of the artificial ulcer after ESD, and presence/absence of remnant/relapse in the operated part and level of healing of the artificial ulcer were evaluated.

### Statistical analysis

SAS software (SAS Institute, Cary, NC, USA) was used for analyses. The efficacy rate of the primary outcome measure was evaluated as the difference between the SA and SH groups; the 95% confidence interval (CI) was calculated. If the lower confidence limit of the calculated CI was above the non‐inferiority margin (−16.8%), non‐inferiority was demonstrated. For secondary outcome measures, we examined the equilibrium of distribution among groups by two‐sample Wilcoxon test with ranked categorization data. Regarding non‐ranked categorization data, Fisher's exact test was used for examining the equilibrium of distribution among the groups. Age, height and bodyweight were compared by Student's *t*‐test. The significance level of the two‐tailed test was 5% on both sides.

### Sample size

In 0.4% SH study, with similar primary outcome measures for endoscopic resection during treatment of patients with gastric intramucosal tumor, the efficacy rate was 90.3% and 56.7% for 0.4% SH and physiological saline, respectively.^20^ When the non‐inferiority margin would be set as 16.8%, which is half of the difference in efficacy rate between 0.4% SH and physiological saline, the number of patients required for verifying non‐inferiority would be 59 patients/group in this study (single‐tailed significance level α = 2.5%, statistical power 1−β = 80%). In anticipation of 10% patients being excluded from each group, each group should comprise 65 patients (130 patients in total).

## Results

**Figure 1:**
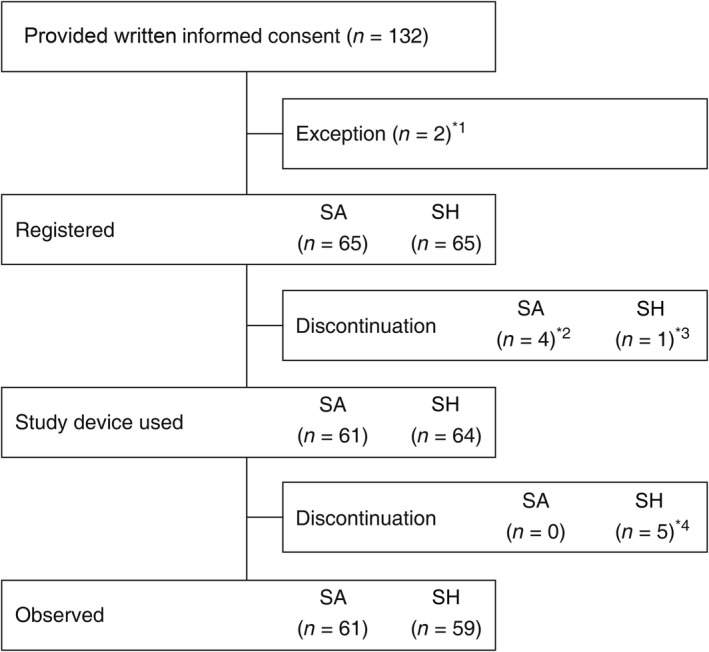
Flow chart of the study. *^1^Excluded for the following reasons: one patient for withdrawal of consent and one patient for meeting exclusion criteria. *^2^Discontinued for the following reasons: two patients for withdrawal of consent and two patients for meeting exclusion criteria. *^3^Discontinued because of no neoplastic lesions by endoscopy. *^4^Discontinued for the following reasons: one patient for occurrence of an adverse event during the study, one patient for treatment of another lesion, two patients for performance of other treatments because of deeper submucosal invasion and one patient for no neoplastic lesions by histopathology.

Figure 1 shows a flow chart of the study.

Of the 130 patients registered in this study, 125 patients (SA group, *n* = 61; SH group, *n* = 64) received a submucosal injection solution and represented the group to be analyzed for safety. The full analysis set included 122 patients only (SA group, *n* = 60; SH group, *n* = 62) because three patients did not have any neoplastic lesion in the resected specimen after ESD.

Characteristics of both groups are summarized in Table 2. The neoplastic lesion sites were located as follows: esophagus in 15.0% (9/60) and 16.1% (10/62) in the SA and SH groups, respectively; and stomach in 85.0% (51/60) and 83.9% (52/62) in the SA and SH groups, respectively.

**Table 2:** Characteristics of the 122 patients in the full analysis set

Characteristic	SA (*n* = 60)	SH (*n* = 62)	*P*
Age (years)			
Mean (SD)	68.4 (6.9)	68.7 (7.7)	0.786^a^
Range	46–80	42–80	
Sex, *n* (%)			
Male	47 (78.3)	48 (77.4)	1.000^b^
Female	13 (21.7)	14 (22.6)	
Height (cm)			
Mean (SD)	163.7 (7.2)	164.2 (8.0)	0.728^a^
Range	145.0–176.6	144.5–184.7	
Bodyweight (kg)			
Mean (SD)	63.5 (11.1)	62.2 (10.0)	0.492^a^
Range	41.3–84.9	38.7–82.9	
Smoking, *n* (%)	12 (20.0)	9 (14.5)	0.478^b^
Drinking alcohol, *n* (%)	36 (60.0)	40 (64.5)	0.709^b^
Gastrointestinal location, *n* (%)			
Esophagus	9 (15.0)	10 (16.1)	1.000^b^
Stomach	51 (85.0)	52 (83.9)	

a Student's *t*‐test.

b Fisher's exact test.

SA, sodium alginate; SD, standard deviation; SH, sodium hyaluronate.

Histological types identified by biopsy before ESD for esophageal mucosal neoplastic lesions were distributed as follows in the SA group: intraepithelial neoplasia in 11.1% (1/9), malignant epithelial neoplasm in 55.6% (5/9) and biopsy in participating institutions was not performed in 33.3% (3/9). In the SH group, intraepithelial neoplasia was observed in 20.0% (2/10), malignant epithelial neoplasm in 40.0% (4/10) and biopsy in participating institutions was not performed in 40.0% (4/10) (Table 3).

**Table 3:** Preoperative endoscopic findings of esophageal intramucosal neoplasm

	SA (*n* = 9)	SH (*n* = 10)	*P* a
Histological type, *n* (%)^b^			
Intraepithelial neoplasia			1.000
Squamous intraepithelial neoplasia	1 (11.1)	2 (20.0)	
Malignant epithelial neoplasm			
Squamous cell carcinoma	5 (55.6)	4 (40.0)	
No biopsy done	3 (33.3)	4 (40.0)	
Neoplasm location, *n* (%)			
Upper thoracic esophagus	1 (11.1)	4 (40.0)	0.164
Middle thoracic esophagus	6 (66.7)	6 (60.0)	
Lower thoracic esophagus	2 (22.2)	0 (0.0)	
Macroscopic classification, *n* (%)			
0–II b	2 (22.2)	3 (30.0)	1.000
0–II c	7 (77.8)	7 (70.0)	
Neoplasm size (mm), *n* (%)			
5–9	1 (11.1)	2 (20.0)	1.000
10–14	1 (11.1)	2 (20.0)	
15–20	7 (77.8)	6 (60.0)	

a Fisher's exact test.

b Histological type in biopsy with preoperative endoscopy.

SA, sodium alginate; SH, sodium hyaluronate.

Histological types identified by biopsy before ESD in gastric mucosal neoplastic lesions were distributed as follows in the SA group: benign epithelial neoplasia (adenoma) in 2.0% (1/51), malignant epithelial tumor in 60.8% (31/51), others in 2.0% (1/51) and biopsy in participating institutions was not performed in 35.3% (18/51). In the SH group, benign epithelial neoplasia (adenoma) was observed in 1.9% (1/52), malignant epithelial tumor in 59.6% (31/52), others in 3.8% (2/52) and biopsy in participating institutions was not performed in 34.6% (18/52) (Table 4).

**Table 4:** Preoperative endoscopic findings of gastric intramucosal neoplasm

	SA (*n* = 51)	SH (*n* = 52)	*P* a
Histological type, *n* (%)^b^			
Benign epithelial neoplasia			0.731
Adenoma	1 (2.0)	1 (1.9)	
Malignant epithelial neoplasm			
Tub^c^			
Tub1^d^	27 (52.9)	30 (57.7)	
Tub2^e^	4 (7.8)	1 (1.9)	
Other	1 (2.0)^f^	2 (3.8)^g^	
No biopsy done	18 (35.3)	18 (34.6)	
Neoplasm location, *n* (%)			
Upper third	6 (11.8)	7 (13.5)	0.890
Middle third	19 (37.3)	21 (40.4)	
Lower third	26 (51.0)	24 (46.2)	
Section of wall, *n* (%)			
Lesser curvature	21 (41.2)	21 (40.4)	0.942
Greater curvature	11 (21.6)	13 (25.0)	
Anterior curvature	9 (17.6)	10 (19.2)	
Posterior curvature	11 (21.6)	9 (17.3)	
Macroscopic classification, *n* (%)			
0–I	0 (0.0)	1 (1.9)	0.472
0–IIa	16 (31.4)	22 (42.3)	
0–IIc	32 (62.7)	28 (53.8)	
0–IIa + IIc	2 (3.9)	1 (1.9)	
0–IIb + IIc	1 (2.0)	0 (0.0)	
Neoplasm size (mm), *n* (%)			
5–9	20 (39.2)	17 (32.7)	0.786
10–14	22 (43.1)	24 (46.2)	
15–20	9 (17.6)	11 (21.2)	

a Fisher's exact test.

b Histological type in biopsy with preoperative endoscopy.

c Tubular adenocarcinoma.

d Well differentiated.

e Moderately differentiated.

f One patient whose histological classification was unknown.

g One patient who was unknown whether well differentiated or moderately differentiated, and one patient evaluated as erosive gastritis with regenerative atypia.

SA, sodium alginate; SH, sodium hyaluronate.

Regarding the size of resected specimen and depth of invasion assessed on pathological examination after ESD, no significant differences were noted between the groups (Table 5).

**Table 5:** Size of resected specimen and depth of invasion on pathological examination

	SA	SH	*P*
Esophageal lesion	(*n* = 9)	(*n* = 10)	
Size of resected specimen (mm)			
Mean (SD)	26.6 (5.1)	27.2 (6.3)	0.810^a^
Depth of invasion, *n* (%)			
pT1a‐EP	5 (55.6)	2 (20.0)	0.053^b^
pT1a‐LPM	2 (22.2)	7 (70.0)	
pT1a‐MM	2 (22.2)	0 (0.0)	
Unable to determine	0 (0.0)	1 (10.0)^c^	
Gastric lesion	(*n* = 51)	(*n* = 52)	
Size of resected specimen (mm)			
Mean (SD)	34.2 (6.1)	35.3 (8.1)	0.447^b^
Depth of invasion, *n* (%)			
pT1a	47 (92.2)	43 (82.7)	0.276^b^
pT1b	3 (5.9)	8 (15.4)	
Unable to determine	1 (2.0)^d^	1 (1.9)^d^	

a Student's *t*‐test.

b Fisher's exact test.

c For squamous intraepithelial neoplasia.

d For adenoma.

SA, sodium alginate; SD, standard deviation; SH, sodium hyaluronate.

### Efficacy evaluation

Table 6 shows a summary of efficacy evaluation results in this study. Regarding the primary outcome measure, comprehensive evaluation of en bloc complete resection in ESD and additional injection resulted in an efficacy rate of 91.7% (55/60) and 88.7% (55/62) in the SA and SH groups, respectively; the difference in the efficacy rate was 3.0% (95% CI of difference, −9.35% to 15.26%). Because the lower confidence limit of the 95% CI was −9.35%, which is higher than the non‐inferiority margin of −16.8%, non‐inferiority of SA against SH was demonstrated (*P* < 0.001). En bloc complete resection rates in the SA and SH groups were 100.0% (60/60) and 96.8% (60/62), respectively; no difference between the groups was noted (*P* = 0.496). Percentages of patients who received no or one additional injection because of mucosal elevation disappearance in the SA and SH groups were 91.7% (55/60) and 90.3% (56/62), respectively; no significant difference was noted between the groups (*P* = 0.994). Regarding secondary outcome measures, mucosal resection ease using submucosal injection solution was evaluated as ‘extremely easy’/‘easy’ by 85.0% (51/60) and 85.5% (53/62) in the SA and SH groups, respectively; no significant difference was noted between the groups. Moreover, regarding mucosal elevation shape, time required for mucosal resection and volume of submucosal injection solution used, no significant differences were noted between the groups.

**Table 6:** Summary of primary and secondary outcome measurement results

	SA (*n* = 60)	SH (*n* = 62)	*P*
Primary outcome measure			
Efficacy rate, % (*n*)	91.7 (55)	88.7 (55)	<0.001^a^
En bloc complete resection	100.0 (60)	96.8 (60)	0.496^b^
Additional counts of injection			
0	83.3 (50)	83.9 (52)	0.994^c^
1	8.3 (5)	6.5 (4)	
≥2	8.3 (5)	9.7 (6)	
Secondary outcome measures			
Mucosal resection ease, % (*n*)			
Extremely easy	38.3 (23)	33.9 (21)	0.678^c^
Easy	46.7 (28)	51.6 (32)	
Ordinary	13.3 (8)	9.7 (6)	
Difficult	1.7 (1)	4.8 (3)	
Mucosal elevation shape, % (*n*)			
Steep	66.7 (40)	59.7 (37)	0.426^c^
Gentle elevation	33.3 (20)	40.3 (25)	
No elevation	0.0 (0)	0.0 (0)	
Unable to determine	0.0 (0)	0.0 (0)	
Time required for mucosal resection (min)			
Mean (SD)	39.2 (26.4)	36.2 (22.4)	0.847^c^
Injection volume (mL)			
Mean (SD)	23.3 (11.7)	24.5 (11.6)	0.506^c^

a Hypothesis test that the lower limit of 95% confidence interval (CI) of the difference in efficacy rate does not fall below the non‐inferiority margin (difference in the efficacy rate: 3.0%; 95% CI, −9.35% to 15.26%; non‐inferiority margin, −16.8%).

b Fisher's exact test.

c Wilcoxon two‐sample test.

SA, sodium alginate; SD, standard deviation; SH, sodium hyaluronate.

Table 7 shows exploratory results of the relationship between endoscopically measured tumor size and efficacy rate in the gastric mucosa. For 15–20‐mm tumors, the efficacy rate was 100.0% (9/9) and 54.5% (6/11) in the SA and SH groups, respectively, demonstrating a significantly higher efficacy rate (*P* = 0.038) for the SA group. However, en bloc complete resection rates in the SA and SH groups were 100.0% (9/9) and 90.9% (10/11), respectively; no significant difference was noted between the groups (*P* = 1.000; Table 8). The proportion of patients who did not require additional injection was 88.9% (8/9) and 54.5% (6/11) in the SA and SH groups, respectively; no significant difference was noted between the groups (*P* = 0.075).

**Table 7:** Efficacy rate for gastric intramucosal neoplasm size and location

	SA	SH	*P* a
Neoplasm size (mm)			
5–9	95.0% (19/20)	88.2% (15/17)	0.584
10–14	86.4% (19/22)	100.0% (24/24)	0.101
15–20	100.0% (9/9)	54.5% (6/11)	0.038
Neoplasm location			
Upper third	83.3% (5/6)	85.7% (6/7)	1.000
Middle third	94.7% (18/19)	95.2% (20/21)	1.000
Lower third	92.3% (24/26)	79.2% (19/24)	0.239

a Fisher's exact test.

Efficacy rate was defined as the percentage of en bloc complete resection that required an additional injection number of 0 or 1.

SA, sodium alginate; SH, sodium hyaluronate.

**Table 8:** En bloc complete resection and additional injections for gastric intramucosal neoplasm size

	SA	SH	*P*
Neoplasm size of 5–9 mm	(*n* = 20)	(*n* = 17)	
En bloc complete resection, % (*n*)	100.0 (20)	94.1 (16)	0.460^a^
Additional counts of injection, % (*n*)			
0	90.0 (18)	82.4 (14)	0.457^b^
1	5.0 (1)	5.9 (1)	
≥2	5.0 (1)	11.8 (2)	
Neoplasm size of 10–14 mm	(*n* = 22)	(*n* = 24)	
En bloc complete resection, % (*n*)	100.0 (22)	100.0 (24)	1.000^b^
Additional counts of injection, % (*n*)			
0	81.8 (18)	95.8 (23)	0.117^b^
1	4.5 (1)	4.2 (1)	
≥2	13.6 (3)	0.0 (0)	
Neoplasm size of 15–20 mm	(*n* = 9)	(*n* = 11)	
En bloc complete resection, % (*n*)	100.0 (9)	90.9 (10)	1.000^b^
Additional counts of injection, % (*n*)			
0	88.9 (8)	54.5 (6)	0.075^b^
1	11.1 (1)	9.1 (1)	
≥2	0.0 (0)	36.4 (4)	

a Fisher's exact test.

b Wilcoxon 2‐sample test.

SA, sodium alginate; SH, sodium hyaluronate.

### Adverse events

Adverse events were evaluated in the 125 patients who received a submucosal injection solution (Table 9). The adverse event rate in this study was 36.1% (22/61) and 34.4% (22/64) in the SA and SH groups, respectively. Regarding adverse events specific to ESD, postprocedural hemorrhage was observed at a frequency of 0% (no patient) and 7.8% (5/64) in the SA and SH groups, respectively, whereas perforation was observed at a frequency of 1.6% (1/61) and 1.6% (1/64) in the SA and SH groups, respectively. Six adverse events for which a causal relationship with the submucosal injection solution could not be ruled out, namely anemia, upper abdominal pain, nausea, vomiting, pyrexia and procedural pain, occurred in five patients in the SA group, corresponding to a rate of 8.2%. Three events of esophageal pain, back pain and abdominal pain were noted in three patients in the SH group, corresponding to a rate of 4.7%. All adverse reactions in both groups disappeared without treatment or after drug administration. The incidence of serious adverse events was 4.9% (3/61) and 3.1% (2/64) in the SA and SH groups, respectively; while no cases resulted in mortality or mortality risk, hospitalization or hospitalization duration prolongation was unavoidable for all serious adverse events. There was no significant difference between the groups in the blood sampling test performed preoperatively and 2 weeks postoperatively (data not shown).

**Table 9:** Adverse events related to submucosal injection solution or ESD

Variable	SA (*n* = 61)	SH (*n* = 64)
Patients with adverse events, *n* (%)	22 (36.1)	22 (34.4)
Adverse events occurring in ≥5% of treatment groups, *n* (%)		
Nausea	5 (8.2)	3 (4.7)
Postprocedural hemorrhage	0 (0.0)	5 (7.8)
Procedural pain	4 (6.6)	0 (0.0)
Adverse events specific to ESD, *n* (%)		
Postprocedural hemorrhage	0 (0.0)	5 (7.8)
Perforation	1 (1.6)	1 (1.6)
Patients with serious adverse events, *n* (%)	3 (4.9)^a^	2 (3.1)^b^
Adverse event related to submucosal injection solution, *n* (%)	5 (8.2)^c^	3 (4.7)^d^

a Three patients with hematemesis, perforation, occipital neuralgia and spinal osteoarthritis.

b Two patients with postprocedural hemorrhage.

c Five patients with vomiting, nausea, procedural pain, pyrexia, anemia and upper abdominal pain (there was no significant difference from the ratio of SH group, *P* = 0.423, χ^2^‐test).

d Three patients with abdominal pain, esophageal pain, and back pain.

ESD, endoscopic submucosal dissection; SA, sodium alginate; SH, sodium hyaluronate.

### Endoscopy

Endoscopic examination was performed at 8 weeks after ESD in 121 patients (SA group, *n* = 61; SH group, *n* = 60, including one patient who was discontinued). At 8 weeks after ESD, none of the neoplastic lesions remained; local recurrence was not observed in either group. The cure (state in which the surface of the ulcer was repaired with regenerating epithelium) rate of artificial ulcers in the SA and SH groups at 8 weeks after ESD was 88.5% (54/61) and 95.0% (57/60), respectively; no significant difference was noted between the groups (*P* = 0.078, data not shown).

## Discussion

Currently, gastric ESD has been established as a standard procedure in Japan in terms of reliability and curability with high en bloc resection rate.^21^, ^22^, ^23^, ^24^ Regarding esophageal ESD, circumferential resection has become possible with countermeasures for stenosis. In addition, because early detection has become possible with narrowband imaging, esophageal ESD is becoming widespread.^25^

However, according to the latest gastric cancer treatment guidelines published after the end of this study,^26^ the tumor size criterion is no longer applied to differentiated carcinoma localized in the mucosa with no ulcer findings, and differentiated carcinoma of 3 cm or less localized in the mucosa with ulcer findings is now defined as a lesion for which ESD is absolutely indicated for. Therefore, forming and maintaining a sufficient elevation of the mucosa by a submucosal injection solution remain even more important for ESD safety and efficacy.

In this study, we compared the efficacy and safety of 0.6% SA with those of 0.4% SH, as a control, in ESD performed on patients with a neoplastic lesion localized within the esophagus and gastric mucosa.

As a result, SA forms a steep elevation at the lesion site, and it was verified that maintainability is not inferior to that of SH. However, in larger gastric lesions, namely 15–20‐mm tumors, the efficacy rate of the SA group was significantly higher than that of the SH group. In this study, the ability to maintain lesion elevation by injection was evaluated based on the number of additional injections. For 15–20‐mm tumors, the proportion of patients who did not require additional injection was not significantly different between the SA and SH groups, but it tended to be lower in the SA group. Kusano *et al*.^16^ reported that mucosal elevation formed by injection of 0.6% SA was significantly higher than that formed by 0.4% SH over 30 min after injection in a test system using excised porcine stomach. Similarly, we observed a significant difference in the efficacy rate, although the number of patients in this tumor size subgroup was small. No significant difference was observed between the SA and SH groups regarding the number of additional injections per tumor size groups; however, SA was demonstrated to be capable of maintaining mucosal elevation irrespective of the tumor size.

In this study, the number of patients was set for evaluating efficacy; safety evaluation was not conducted based on statistical analysis because the number of patients was not sufficient for complete safety evaluation. However, the formation and maintenance effect of SA mucosal elevation is at least as good as that of SH, providing an adequate safety margin between the mucosal and muscular layers. This safety margin may lower hemorrhage and perforation risk peculiar to ESD.

### Limitations

Because the form of SA and SH differ, the design of this study was single blind rather than double blind, and although an information bias cannot be denied, the authors determined that there was no significant influence on the evaluation of efficacy.

### Conclusion

In Japan, 0.4% SH is the only commercially approved formulation for submucosal injection during ESD, but the results of this study may expand submucosal injection solution options in clinical practice.

## Conflicts of Interest

The funding for this study was provided by Kaigen Pharma Corporation, Osaka, Japan, which is to receive approval for marketing of the medical device from the Ministry of Health Labor, and Welfare of Japan. The funding source had no role in the design, practice or analysis of this study. Kaigen Pharma Corporation contracted and paid all hospitals on the basis of good clinical practice.

## Author Contribution

Conception and design: N.U. Analysis and interpretation of data: I.O., Y.S., H.O., J.F., N.M., K.O., N.Y., T.Y., H.T., M.I. and S.K. Drafting of the article: M.S. Critical revision of the article for important intellectual content: N.U. Final approval of the article: N.U.
